# A Comparative Study on the Psychological Health of Frontline Health Workers in Wuhan Under and After the Lockdown

**DOI:** 10.3389/fpsyt.2021.701032

**Published:** 2021-06-21

**Authors:** Xiuli Qiu, Yan Lan, Jinfeng Miao, Hui Wang, He Wang, Jianhong Wu, Guo Li, Xin Zhao, Ziqin Cao, Junhua Mei, Wenzhe Sun, Zhou Zhu, Suiqiang Zhu, Wei Wang

**Affiliations:** ^1^Department of Neurology, Tongji Medical College, Tongji Hospital, Huazhong University of Science and Technology, Wuhan, China; ^2^Nursing Department, Tongji Medical College, Tongji Hospital, Huazhong University of Science and Technology, Wuhan, China; ^3^Department of Medical Affair, Tongji Medical College, Tongji Hospital, Huazhong University of Science and Technology, Wuhan, China; ^4^Department of Gastrointestinal Surgery, Tongji Medical College, Tongji Hospital, Huazhong University of Science and Technology, Wuhan, China; ^5^Department of Chemistry, Emory University, Atlanta, GA, United States; ^6^Department of Neurology, Wuhan First Hospital, Wuhan, China

**Keywords:** COVID-19, front-line healthcare workers, depressive symptoms, anxiety symptoms, stress

## Abstract

**Background:** The coronavirus disease-2019 (COVID-19) outbreak and a 3-month lockdown of Wuhan may have had a long-term impact on the mental health of frontline healthcare workers (HWs). However, there is still a lack of comparative studies on the mental health of front-line HWs in the initial phase of the lockdown and 1 month after the lifting of the lockdown.

**Methods:** We recruited 1717 HWs during the initial phase of the lockdown and 2214 HWs 1 month after the lifting of the lockdown, and their baseline characteristics and psychiatric health in these two phases were compared. Furthermore, Pearson's Chi-square test and multivariate logistic regression analysis were used to determine the possible risk factors associated with depressive symptoms in the front-line HWs.

**Results:** Compared with the initial phase of the lockdown, the proportion of HWs with anxiety symptoms and stress decreased, while the proportion of HWs with depressive symptoms increased a month after the lifting of the lockdown. Male sex, exercise habit, comorbidities, and having family members or relatives with suspected or confirmed COVID-19 infection were significantly related to the increased incidence of depressive symptoms during the initial phase of the lockdown. Comorbidities, negative effect of media coverage, working >4 days a week, lower annual household income, and deteriorating relationships with family members were associated with depressive symptoms a month after the lifting of the lockdown.

**Conclusion:** The increased proportion of HWs with depressive symptoms 1 month after the lifting of the lockdown suggested that mental health of front-line HWs should be a top-priority issue, not only during, but also after the pandemic.

## Introduction

The coronavirus disease-2019 (COVID-19) outbreak that reported in Wuhan in December 2019 quickly attracted worldwide attention (1), and stringent control measures were adopted to stop the spread of the outbreak. On January 23rd, 2020, Wuhan officials announced a lockdown in the city, and subsequently suspended all public transportation and placed restrictions on most activities and movements (2). After 3 months of lockdown, public transportation was resumed on April 8 and economic activity started again on a large scale (3). The strict lockdown brought the infection under control in the shortest possible time (4). However, apart from the huge social cost, this outbreak and strict social distancing measures may have a long-term impact on the mental health of the people in Wuhan (5), and even a potential rebound effect with psychological manifestations when the imminent threat of COVID-19 subsides.

When encountering this unexpected and unknown disease, healthcare workers (HWs) experienced increased workloads (6), increased risk of exposure and infection (7), changes in job position and schedules, inadequate availability of personal protective equipment, discrimination, and isolation. Previous studies conducted by us and other authors have shown that during the outbreak of COVID-19, high rates of psychiatric morbidity were found in HWs (8–11). The effects of the COVID-19 pandemic on the long-term mental health of the population have gradually emerged over time (12), and the mental health effects of the COVID-19 pandemic on adolescents, the elderly, and the general population have been reported (13, 14). However, there is still a lack of comparative studies on HWs' mental health in the initial phase of the lockdown and 1 month after the lifting of the lockdown during the COVID-19 outbreak.

Wuhan is a suitable place to study the difference in mental health of frontline HWs between the initial outbreak of COVID-19 and 1 month after the lifting of the lockdown. Tongji Hospital is the major hospital that was responsible for managing severe COVID-19 patients in Wuhan. The administration continually dispatched nearly 3,000 HWs and gradually increased the hospital's capacity by adding 2,000 hospital beds to treat severe COVID-19 patients. We conducted two questionnaires in Tongji Hospital in the initial phase of the lockdown (2 weeks after lockdown) and more than 1 month after the lifting of the lockdown during the COVID-19 outbreak in Wuhan, because there was a great change in the life and work patterns of HWs. Therefore, a cross-sectional study was designed to evaluate the psychological health of our study participants when the COVID-19 outbreak escalated, as well as after it subsided, in order to compare the psychiatric morbidity, and explore the factors related to depressive symptoms between the two phases.

## Materials and Methods

### Study Design and Participants

In this single-center cross-sectional comparative study, all frontline HWs recruited were the employees of Tongji Hospital, including all doctors, nurses, and medical technicians who directly provided medical services to the patients that had a confirmed or suspected COVID-19 infection. Frontline HWs with a history of psychiatric disease were excluded from the study. An online anonymous questionnaire survey was used for acquiring information. The survey was conducted twice, once in the initial phase of the lockdown of the COVID-19 outbreak from February 8 to February 15, 2020, 2 weeks after the Wuhan lockdown, and then more than 1 month after the lifting of the lockdown between May 27 and June 7, 1 month after the eradication of COVID-19 from Wuhan (15), work and production were resumed in an orderly fashion. Each participant could answer the questionnaire only once per survey.

The structured questionnaire consisted of four main components: online informed consent, sociodemographic characteristics, perceptions regarding the COVID-19 threat, and the rating scales to determine the participants' psychological well-being, which included the Impact of Event Scale-Revised Questionnaires (IES-R), Patient Health Questionnaire-9 (PHQ-9), and Generalized Anxiety Disorder 7-item (GAD-7) questionnaire. Since working overtime, media coverage, and family relationships during the COVID-19 outbreak may affect the frontline HWs mental health as well, changes in relationships with family members, media's influence on the participant's emotions, and the frequency and duration of work during the outbreak were added to the questionnaire of the survey conducted a month after the lifting of the lockdown. An e-questionnaire was administered *via* the WJX online survey platform (https://www.wjx.cn/), and the data were collected *via* WeChat (Tencent Holdings Ltd., Shenzhen, Guangdong province, China). The online informed consent was obtained from all the participants. The study was approved by the institutional ethics board of Tongji Hospital (ID: TJ-C20200129) and conforms to the principles stated in the Declaration of Helsinki.

### Measures

The included sociodemographic characteristics and the definition of exercise habit were the same as stated in our previous study (11). We used the following six items to assess the perceptions regarding the COVID-19 threat: (1) Have you ever thought of resigning because of the COVID-19 outbreak? (2) Have you worried about life-threatening illness from infection? (3) Do you feel that families and friends have avoided contact with you because of your work? (4) Are you satisfied with the full measures (11) taken by the departments for preventing the dissemination of nosocomial infection? (5) Are you satisfied with your work shift arrangement? (6) In the past 2 weeks, have you had at least one suicidal thought?

In order to understand the psychological impact caused by the COVID-19 outbreak, the PHQ-9, and GAD-7 scales were used to measure the symptom severity of depression, and anxiety, respectively. In addition, we used IES-R (a 22-item self-reported scale describing avoidance, intrusion, and hyperarousal symptoms after a traumatic event) to assess the subjective stress caused by the specific event of COVID-19 (16–19). The total scores of these measuring tools were categorized as follows: PHQ-9, normal (0–4), mild (5–9), moderate (10–14), and severe (15–27) depression; GAD-7, normal (0–4), mild (5–9), moderate (10–14), and severe (15–21) anxiety; and IES-R, normal (0–8), mild (9–25), moderate (26–43), and severe (44–88) stress (20–22). These symptoms were classified based on the values already established in the literature, and the scores that met or exceeded the threshold of the “mild” category, were defined as the depressive or, anxiety symptoms, or stress.

### Statistical Analysis

Data analysis was performed using SPSS V.22.0 (IBM Corp., Armonk, NY, USA). After receiving the data, continuous variables were first categorized as categorical variables, and then all variables were displayed as counts and percentages. Variables with *p* < 0.05 according to the Pearson's Chi-square test were subjected to multivariate logistic regression analysis with a stepwise backward selection method. In order to determine the potential risk factors for depressive symptoms a month after the lifting of the lockdown, a multivariate logistic regression analysis was performed and the relationship between the related factors and outcomes was expressed as odds ratio (OR) with 95% confidence interval (CI).

## Results

### Sociodemographic Characteristics of the Frontline HWs in the Initial Phase of the Lockdown and 1 Month After the Lifting of the Lockdown

**Figure 1:**
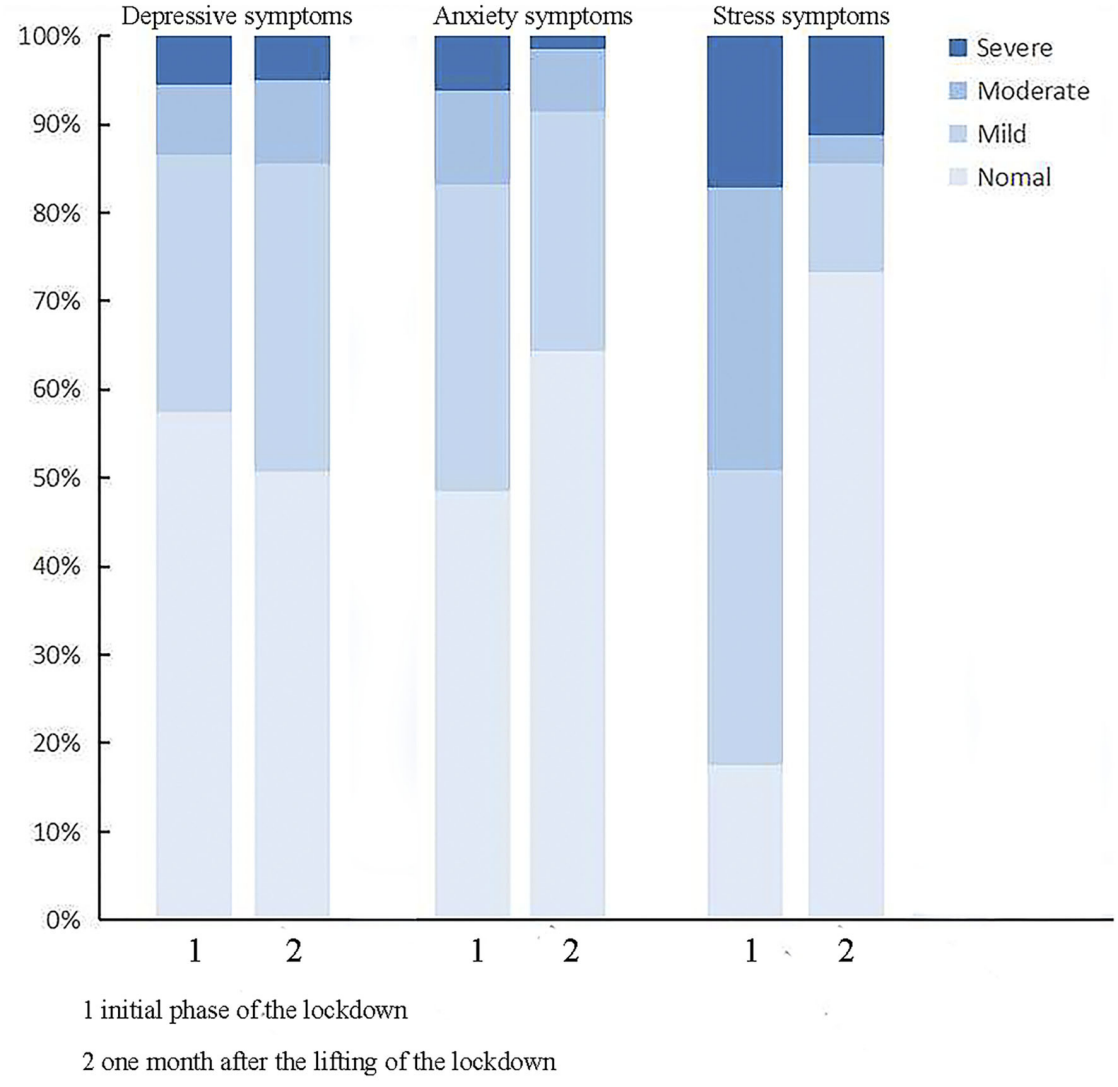
Distribution of severity categories of depressive symptoms, anxiety symptoms, and stress in the initial phase of the lockdown and 1 month after the lifting of the lockdown of COVID-19.

Two surveys were conducted on 3,110 frontline HWs in Tongji Hospital. In the initial phrase, 1,717 questionnaires were collected (response rate was 55.2%) and 2,214 questionnaires were collected 1 month after the lifting of the lockdown (response rate was 71.2%). In the initial phase of the lockdown, 730 (42.5%) individuals developed depressive symptoms, 883 (51.4%) had anxiety symptoms and 1,417 (82.5%) had stress. Data collected a month after the lifting of the lockdown showed that 1,089 (49.2%) frontline HWs developed depressive symptoms, 784 (35.4%) had anxiety symptoms, and 590 (26.6%) experienced stress (Figure 1). There were no significant difference between the two respondent groups in terms of their baseline characteristics, which included age, annual household income, marital status, educational level, years of working, parental status, and families or relatives with suspected or confirmed COVID-19 infection (Table 1).

**Table 1:** Sociodemographic characteristics of front-line health workers dealing with the COVID-19 in two phases.

**Characteristics**	**Initial phase of the lockdown (*N* = 1,717), *n* (%)**	**1 month after the lockdown was lifted (*N* = 2,214), *n* (%)**	***P*-value**
Age			0.323
19–29 years	695 (40.5)	867 (39.2)	
30–49 years	998 (58.1)	1,325 (59.8)	
>49 years	24 (1.4)	22 (1.0)	
Gender			0.008
Men	281 (16.4)	296 (13.4)	
Women	1,436 (83.6)	1,918 (86.6)	
Occupation			<0.001
Doctor	325 (18.9)	420 (19.0)	
Nurse	1,226 (71.4)	1,751 (79.1)	
Medical technician	166 (9.7)	43 (1.9)	
Annual household income			0.197
>200 thousand Yuan	616 (35.9)	839 (37.9)	
100–200 thousand Yuan	835 (48.6)	1,072 (48.4)	
30–100 thousand Yuan	266(15.5)	303 (13.7)	
Exercise habit	279 (16.2)	261 (11.8)	<0.001
Parent status			0.429
No child	718 (41.8)	955 (43.1)	
One child	805 (46.9)	993 (44.9)	
Two or more children	194 (11.3)	266 (12.0)	
Marital status			0.881
Married	1,089 (63.4)	1,401 (63.3)	
Unmarried	591 (34.4)	770 (34.8)	
Divorced/widowed/separated	37 (2.2)	43 (1.9)	
Education			0.058
Bachelor degree or below level	1,325 (77.2)	1,764 (79.7)	
Master degree or higher level	392 (22.8)	450 (20.3)	
Professional title level			0.008
Junior	1,050 (61.2)	1,452 (65.6)	
Intermediate	569 (33.1)	632 (28.5)	
Senior	98 (5.7)	130 (5.9)	
Years of working			0.193
<2 years	111 (6.5)	174 (7.9)	
2–5 years	660 (38.4)	794 (35.9)	
6–10 years	538 (31.3)	719 (32.5)	
>10 years	408 (23.8)	527 (23.8)	
Past medical history			<0.001
In good health	1,471 (85.7)	1,992 (90.0)	
Have comorbidities	246 (14.3)	222 (10.0)	
Smoking	43 (2.5)	21 (0.9)	<0.001
Drinking	150 (8.7)	61 (2.8)	<0.001
Families or relatives suspected or confirmed	220 (12.8)	263 (11.9)	0.376

### Perceptions Regarding the COVID-19 Threat in the Initial Phase of the Lockdown and 1 Month After the Lifting of the Lockdown

As shown in Table 2, compared to a month after the lifting of the lockdown, more frontline HWs had considered resigning due to the COVID-19 outbreak (14.8 vs. 5.1%, *p* < 0.001), had worried about life-threatening illness once infected (63.2 vs. 29.4%, *p* < 0.001) and had felt that their family members and friends were avoiding contact due to their job (22.0 vs. 11.6%, *p* < 0.001) in the initial phase of the lockdown. However, they were more satisfied with their work shift arrangement (75.2 vs. 71.9%, *p* < 0.001) and the full measures taken by all the departments for avoiding nosocomial infection (83.9 vs. 81.5%, *p* = 0.049) in this phase. While at 1 month after the lifting of the lockdown, more frontline HWs reported having at least one suicidal thought in the last 2 weeks (8.7 vs. 11.2%, *p* = 0.010), and were less satisfied with their work shift arrangement.

**Table 2:** Perceptions of threat of the COVID-19 among front-line HWs dealing with the COVID-19 in two phases.

**Characteristics**	**Initial phase of the lockdown (*N* = 1,717), *n* (%)**	**1 month after the lifting of the lockdown (*N* = 2,214), *n* (%)**	***P*-value**
Have you ever thought of resigning because of the COVID-19 outbreak?	254 (14.8)	114 (5.1)	<0.001
Have you worried about the life-threatening once infected?	1,086 (63.2)	650 (29.4)	<0.001
Do you feel that families and friends have avoided contact with you because of your work?	378 (22.0)	257 (11.6)	<0.001
Are you satisfied with full coverage of all departments for avoiding nosocomial infection?	1,441 (83.9)	1,805 (81.5)	0.049
Are you satisfied with your work shift arrangement?	1,291 (75.2)	1,591 (71.9)	0.019
Have you had at least one suicidal thought in the last 2 weeks?	150 (8.7)	247 (11.2)	0.013

### Depressive and Anxiety Symptoms, and Stress Between the Initial Phase of the Lockdown and 1 Month After the Lifting of the Lockdown

**Figure 2:**
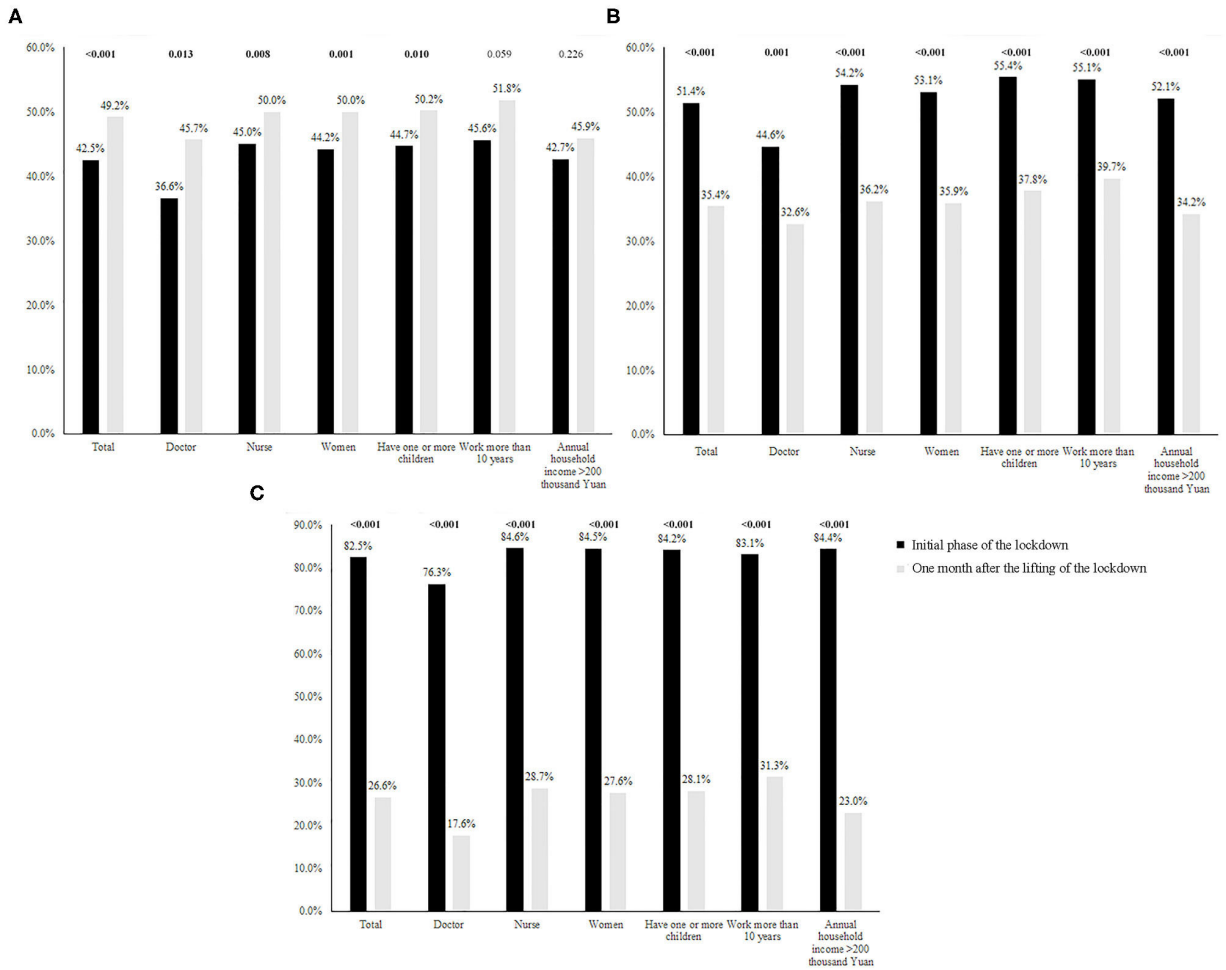
Depressive symptoms, anxiety symptoms, and stress in the initial phase of the lockdown and 1 month after the lifting of the lockdown. **(A)** Depression. **(B)** Anxiety. **(C)** Stress.

In the initial phase of the lockdown, frontline HWs experienced a greater proportion of anxiety symptoms and stress. However, 1 month after the lifting of the lockdown; the proportion of depressive symptoms was higher. Whether for doctors, nurses, women or individuals with children, this trend (more anxiety symptoms and stress in the initial phase of the lockdown and more depressive symptoms 1 month after the lifting of the lockdown) seemed to be consistent. Of note was that for the people with annual household income >200 thousand Yuan and those who had worked longer than 10 years, the increase in the proportion of depressive symptoms was not significant (Figure 2).

### Factors Associated With Depressive Symptoms in the Initial Phase of the Lockdown and 1 Month After the Lifting of the Lockdown

Considering the differences in the proportion of depressive symptoms and average PHQ-9 scores between the two phases, the Pearson's Chi-square test and multivariate logistic regression analysis were used to examine the association between the different variables and depressive symptoms in the two phases. Table 3 shows the difference in the characteristics between the frontline HWs with and without depressive symptoms in the two phases.

**Table 3:** Sociodemographic characteristics associated with depressive symptoms in front-line HWs in two phases.

**Characteristics**	**Initial phase of the lockdown**		***P*-value**	**1 month after the lifting of the lockdown**		***P*-value**
	**Non-depressive symptoms (*N* = 987), *n* (%)**	**Depressive symptoms (*N* = 730), *n* (%)**		**Non-depressive symptoms (*N* = 1,125), *n* (%)**	**Depressive symptoms (*N* = 1,089), *n* (%)**	
Age			0.101			0.287
19–29 years	415 (42.0)	280 (38.4)		456 (40.5)	411 (37.7)	
30–49 years	555 (56.2)	443 (60.7)		660 (58.7)	665 (61.1)	
>49 years	17 (1.7)	7 (1.0)		9 (0.8)	13 (1.2)	
Gender			0.002			0.051
Men	185 (18.7)	96 (13.2)		166 (14.8)	130 (11.9)	
Women	802 (81.3)	634 (86.8)		959 (85.2)	959 (88.1)	
Occupation			0.004			0.283
Doctor	206 (20.9)	119 (16.3)		228 (20.3)	192 (17.6)	
Nurse	674 (68.3)	552 (75.6)		876 (77.9)	875 (80.3)	
Medical technician	107 (10.8)	59 (8.1)		21 (1.9)	22 (2.0)	
Working more than 4 days a week during the outbreak				538 (47.8)	576 (52.9)	0.017
Negative effects of media during the outbreak				231 (20.5)	286 (26.3)	0.001
Annual household income			0.187			0.027
>200 thousand Yuan	353 (35.8)	263 (36.0)		454 (40.4)	385 (35.4)	
100–200 thousand Yuan	468 (47.4)	367 (50.3)		532 (47.3)	540 (49.6)	
30–100 thousand Yuan	166 (16.8)	100 (13.7)		139 (12.4)	164 (15.1)	
Exercise habit	178 (18.0)	101 (13.8)	0.020	144 (12.8)	117 (10.7)	0.134
Parent status			0.073			0.323
No child	435 (44.1)	283 (38.8)		498 (44.3)	457 (42.0)	
One child	441 (44.7)	364 (49.9)		502 (44.6)	491 (45.1)	
Two or more children	111 (11.2)	83 (11.4)		125 (11.1)	141 (12.9)	
The relationship with family members during the outbreak						<0.001
No change				457 (40.6)	415 (38.1)	
Better				607 (54.0)	553 (50.8)	
Worse				61 (5.4)	121 (11.1)	
Marital status			0.496			0.103
Married	617 (62.5)	472 (64.7)		713 (63.4)	688 (63.2)	
Unmarried	346 (35.1)	245 (33.6)		397 (35.3)	373 (34.3)	
Divorced/widowed/separated	24 (2.4)	13 (1.8)		15 (1.3)	28 (2.6)	
Education			0.141			0.084
Bachelor degree or below level	749 (75.9)	576 (78.9)		880 (78.2)	884 (81.2)	
Master degree or higher level	238 (24.1)	154 (21.1)		245 (21.8)	205 (18.8)	
Professional title level			0.686			0.313
Junior	605 (61.3)	445 (61.0)		745 (66.2)	707 (64.9)	
Intermediate	322 (32.6)	247 (33.8)		308 (27.4)	324 (29.8)	
Senior	60 (6.1)	38 (5.2)		72 (6.4)	58 (5.3)	
Years of working			0.021			0.283
<2 years	72 (7.3)	39 (5.3)		95 (8.4)	79 (7.3)	
2–5 years	402 (40.7)	258 (35.3)		418 (37.2)	376 (34.5)	
6–10 years	291 (29.5)	247 (33.8)		358 (31.8)	361 (33.1)	
>10 years	222 (22.5)	186 (25.5)		254 (22.6)	273 (25.1)	
Past medical history			0.010			<0.001
In good health	864 (87.5)	607 (83.2)		1,042 (92.6)	950 (87.2)	
Have comorbidities	123 (12.5)	123 (16.8)		83 (7.4)	139 (12.8)	
Smoking	25 (2.5)	18 (2.5)	0.930	8 (0.7)	13 (1.2)	0.241
Drinking	83 (8.4)	67 (9.2)	0.577	29 (2.6)	32 (2.9)	0.604
Families or relatives suspected or confirmed	105 (10.6)	115 (15.8)	0.002	110 (9.8)	153 (14.0)	0.002

According to the multivariate logistic regression analysis, male sex (OR = 0.70, 95% CI, 0.53–0.92; *p* = 0.010), exercise habit (OR = 0.76, CI, 0.58–0.996; *p* = 0.046), comorbidities (OR = 1.33, CI, 1.01–1.75; *p* = 0.043), and family members or relatives with suspected or confirmed COVID-19 infection (OR = 1.46, CI, 1.10–1.96; *p* = 0.010), were significantly related to the initial phase of the lockdown depressive symptoms. At the same time, feeling a negative effect of media coverage (OR = 1.31; CI, 1.07–1.61; *p* = 0.008), working more than 4 days a week (OR = 1.22, CI, 1.03–1.44; *p* = 0.025), lower annual household income (100–200 thousand Yuan OR = 1.21, CI, 1.01–1.46; *p* = 0.041; 30–100 thousand Yuan OR = 1.49, CI, 1.14–1.95; *p* = 0.004), deteriorating relationships with family members (OR = 1.98, CI, 1.41–2.78; *p* <0.001), comorbidities (OR = 1.70, CI, 1.27–2.28; *p* < 0.001), and family members or relatives with suspected or confirmed COVID-19 infection (OR = 1.41, CI, 1.08–1.84; *p* = 0.013), were significantly associated with depressive symptoms 1 month after the lifting of the lockdown (Table 4).

**Table 4:** Factors associated with depressive symptoms for front-line HWs in two phases of COVID-19.

**Characteristics**	**Subjects with and without depressive symptoms in initial phase of the lockdown**		**Subjects with and without depressive symptoms 1 month after the lifting of the lockdown**	
	**OR (95% CI)**	***P*-value**	**OR (95% CI)**	***P*-value**
Men	0.70 (0.53–0.92)	0.010		
Negative effects of news media during the outbreak			1.31 (1.07–1.61)	0.008
Working more than 4 days a week during the outbreak			1.22 (1.03–1.44)	0.025
Annual household income				0.009
>200 thousand Yuan				Ref
100–200 thousand Yuan			1.21 (1.01–1.46)	0.041
30–100 thousand Yuan			1.49 (1.14–1.95)	0.004
The relationship within families during the outbreak				<0.001
No change				Ref
Better			0.99 (0.83–1.18)	0.890
Worse			1.98 (1.41–2.78)	<0.001
Exercise habit	0.76 (0.58–0.996)	0.046		
Years of working		0.090		
<2 years		Ref		
2–5 years	1.15 (0.75–1.75)	0.528		
6–10 years	1.45 (0.95–2.23)	0.087		
>10 years	1.42 (0.92–2.21)	0.118		
Have comorbidities	1.33 (1.01–1.75)	0.043	1.70 (1.27–2.28)	<0.001
Families or relatives suspected or confirmed	1.46 (1.10–1.96)	0.010	1.41 (1.08–1.84)	0.013

## Discussion

We have an incomplete understanding of the public response and the impact on the regional economy during bio-disasters. This study found that, compared with a previous longitudinal Chinese study (16), the proportion of HWs with anxiety symptoms and stress decreased in the entire cohort as well as in multiple susceptible groups 1 month after the lifting of the lockdown, which may be related to the psychological resilience that acts as a protective factor in minimizing physical and psychological stress (11, 23). As this study showed, the perception of resignation, fear of acquiring life-threatening illness once infected, and feeling that the family members and friends were avoiding contact decreased when the outbreak had been brought under control. In particular, only 17 frontline HWs were infected in Tongji Hospital during the outbreak, which resulted in mainly mild and moderate illness (24). The extremely low first-line HWs infection rate and satisfaction with the protective measures might have also reduced the perception of threat.

In both the initial phase of the lockdown and 1 month after the lifting of the lockdown, comorbidities and family members or relatives with suspected or confirmed COVID-19 infection were the common factors associated with depressive symptoms in this study. However, exercise habit, which has been widely confirmed to reduce depressive symptoms, was associated with depressive symptoms only in the initial phase of the lockdown, probably because the frontline HWs who had an exercise habit were unable to adapt to the change when their exercise routine was interrupted during the outbreak. In addition, depressive symptoms a month after the lifting of the lockdown were related to lower annual household income, the negative effects of media, working more than 4 days a week, and the deterioration of familial relations during the COVID-19 outbreak.

The media is a double-edged sword (25, 26). On one hand, publicity through the media is conducive to the implementation of government-led control measures, real-time reporting of pandemic control situation, building up a heroic image of the frontline HWs in the eyes of the public, and helping to boost the morale of frontline HWs (27). On the other hand, media reports of infections and deaths among the frontline HWs, the rising number of new cases, the shortage of personal protective equipment, and coronavirus conspiracy theories have increased tension, panic and anxiety symptoms among the frontline HWs (28, 29). In addition, the media may overpublicize and praise the frontline HWs in order to motivate them, and broadcasting reports on the advanced deeds of a few frontline HWs will cause psychological disparity among other frontline HWs when the emergency situation has subsided, especially if they may still face adverse circumstances after returning to work (30, 31).

An Australian study which was conducted after 4 weeks of lockdown during the COVID-19 pandemic has shown a link between reduced income and mental health (32). In this study, although the proportion of depressive symptoms was found to increase in most of the subgroups 1 month after the lifting of the lockdown, this increase was not observed in the subgroup with high annual household income (>200 thousand Yuan). However, depressive symptoms were found to be related to lower annual household income a month after the lifting of the lockdown, but not in the initial phase of the lockdown. This may be because high-income groups have a stronger ability to withstand the economic recession caused by the outbreak (33). In response, the Chinese government decided to give additional allowances to the frontline HWs (34).

This study found that 11.1% of the participants with depressive symptoms reported deteriorating family relationships, compared to 5.4% of the participants without depressive symptoms. During the COVID-19 pandemic, due to the high risk of infection, frontline HWs reduced the contact with their families (7, 35), thus leading to the loss of familial support and increased dissatisfaction of the spouse. In addition, normal family activities were put on hold, causing tension between the parents and children (36). It is well known that depressive symptoms may also affect familial relationships (37). In this cross-sectional study, the causal relationship between depressive symptoms and deteriorating family relationships could not be clarified.

A multinational, multicenter study showed that the prevalence of psychological adversity among HWs seems to be predicted by the HWs' medical history and whether they had physical COVID-19 symptoms, while being independent of the burden of COVID-19 cases within each country (38). There is extensive literature regarding the various factors related to mental health during the COVID-19 pandemic such as being single, separated, or widowed; a higher education level (39); a larger family size; loss of job; physical symptoms (40); and being in contact with potential COVID-19 patients were all associated with an increased level of depression, stress, and anxiety (41–43). Social distancing, being female, having chronic conditions, and living in the family with three to five members were associated with lower HRQOL scores (44). The levels of perceived importance of the “Mandatory quarantine and personal protective equipment” measures were inversely associated with having a post-graduate education, working as white-collar workers, and having fixed-term, full-time employment (45).

The universal, long-term impact of this ongoing traumatic event underscores the importance of longitudinal mental health care for HWs (17). Notably, the strict lockdown measures had a negative psychological impact on psychiatric patients during the COVID-19 pandemic (46). In the future, priority should be given to screening people at a high risk of developing symptoms of depression and promoting the delivery of effective mental health services for the individuals who already had psychiatric disorders or who started experiencing psychiatric disorders during the pandemic. Specifically, digital cognitive behavioral therapy has been shown to improve psychiatric symptoms, indicating that it will be helpful to treat symptoms such as insomnia (47). Joint multidisciplinary assessment and care can contribute to maintaining mental health in the wake of the pandemic (48). Additionally, hospitals and governments can work in tandem to convene psychiatrists and mental health associations, thereby organizing expert groups to develop guidelines and public health education articles/videos for both mental health professionals and the public. Governments can also provide online mental health services. Teams of mental health professionals and experts are installed in designated isolation hospitals to provide on-site services.

The development and clinical trials of the COVID-19 vaccine are very mature, and the vaccine is a both safe and effective preventive measure for COVID-19. According to a study, most HWs believed that COVID-19 is a serious disease and were willing to be vaccinated without worrying about the side effects, economic burden, and stigmatization (49). Vaccination can effectively establish population immunity thus avoiding another COVID-19 pandemic as far as possible.

The strengths of this study are as follows. Firstly, this study had a large sample size and enrolled frontline HWs of a hospital in the initial phase of the lockdown of the COVID-19 outbreak and 1 month after the lifting of the lockdown. The larger the sample size, the better the generated statistical effect. Secondly, the frontline HWs targeted in this study were from Tongji Hospital in Wuhan, which was the major hospital responsible for managing the COVID-19 infection in Wuhan. Thus, our research may help hospital administrators to formulate policies to better address mental health problems of the frontline HWs. Just as children and adolescents are encouraged to keep in touch with their peers through social networks, the government can also provide mental health education and information on preventive measures through mass media (50), People can regulate their emotions through proper form and intensity of indoor exercise (51), and the income and subsidies for HWs can be increased appropriately (34).

Nevertheless, some limitations of the study should be considered. First, online survey and self-rating scales instead of diagnostic interviews were used to assess the mental health status of frontline HWs. Second, although the questionnaires were collected anonymously, the questionnaire survey was conducted by the administrative department of the hospital; therefore, frontline HWs' answers may still harbor social expectations, which may lead to bias in the results. Third, without knowledge of the depression/anxiety scores of the respondents before the onset of the pandemic, we cannot rule out pre-existing depression/anxiety symptoms in the respondents and the fact that more than 80% of the participants were female might make the sample unrepresentative. Fourth, because the group with more exposure and a greater workload did not respond to the questionnaire in the initial phase of the lockdown, a differential response rate (55% in the initial phase of the lockdown and 71% 1 month after the lifting of the lockdown) in the two phases could have introduced bias in the results as well. Fifth, although this study surveyed the main hospital which manages COVID-19 patients in Wuhan, results from this study may not be entirely representative of the general medical community due to the single-center nature of this survey. Sixth, this study only looked at the short term effects (1 month) post the lifting of the lockdown; therefore, further comparative studies focusing on longer term effects should be conducted to better understand the effects on HWs. Lastly, causality cannot be established by cross-sectional surveys; therefore, future prospective studies should be conducted to confirm our findings.

## Conclusion

The cross-sectional survey showed that the psychological ramifications of COVID-19 for the frontline HWs could persist long after the pandemic has ended. This study also identified the factors associated with the depressive symptoms in the frontline HWs in the initial phase of the lockdown and 1 month after the lifting of the lockdown. Although the pandemic is subsiding in many countries, the fragility of mental resilience of the frontline HWs in the post-pandemic era may be more pronounced. Understanding the psychological impact of the COVID-19 outbreak among HWs is crucial in guiding policies and psychological interventions to maintain their long-term psychological well-being.

## Data Availability Statement

The raw data supporting the conclusions of this article will be made available by the authors, without undue reservation.

## Ethics Statement

The study was approved by the institutional ethics board of Tongji Hospital (ID: TJ-C20200129), and conforms to the principles stated in the Declaration of Helsinki. The patients/participants provided their written informed consent to participate in this study.

## Author Contributions

YL, JMi, ZZ, SZ, and WW: concept and design. XQ and YL: acquisition, analysis, and interpretation of data. YL and JMi: statistical analysis. SZ and WW: obtained funding. ZZ, SZ, and WW: supervision. All authors drafting of the manuscript, read, and approved the final edition.

## Conflict of Interest

The authors declare that the research was conducted in the absence of any commercial or financial relationships that could be construed as a potential conflict of interest.

## Abbreviations

COVID-19: coronavirus disease-2019
HW: healthcare workers
SARS: severe acute respiratory syndrome
IES-R: Event Scale-Revised Questionnaires
PHQ-9: Patient Health Questionnaire-9
GAD-7: Generalized Anxiety Disorder 7-item
CI: confidence interval
OR: odds ratio.

## References

[1] Li Q Guan X Wu P Wang X Zhou L Tong Y. Early transmission dynamics in Wuhan, China, of novel coronavirus-infected pneumonia. New Engl J Med. (2020) 382:1199–207. 10.1056/NEJMoa2001316 31995857 PMC7121484

[2] Xinhua. China's Wuhan Suspends Public Transportation, Outward Flights, Trains. (2020). Available online at: http://en.people.cn/n3/2020/0123/c90000-9651334.html.

[3] Xinhua. China Demands Unremitting Containment Efforts as Wuhan Lockdown Lifted. (2020). Available online at: http://en.people.cn/n3/2020/0408/c90000-9677077.html

[4] Xinhua. WHO Congratulates Wuhan on Clearing All COVID-19 Cases. (2020). Available online at: http://en.people.cn/n3/2020/0502/c90000-9686432.html

[5] Vindegaard N Benros ME. COVID-19 pandemic and mental health consequences: systematic review of the current evidence. Brain Behav Immun. (2020) 89:531–42. 10.1016/j.bbi.2020.05.048 32485289 PMC7260522

[6] Wu P Fang Y Guan Z Fan B Kong J Yao Z. The psychological impact of the SARS epidemic on hospital employees in China: exposure, risk perception, and altruistic acceptance of risk. Can J Psychiatr Revue Canadienne Psychiatrie. (2009) 54:302–11. 10.1177/070674370905400504 19497162 PMC3780353

[7] Adams JG Walls RM. Supporting the health care workforce during the COVID-19 global epidemic. JAMA. (2020) 323:1439–40. 10.1001/jama.2020.3972 32163102

[8] Lai J Ma S Wang Y Cai Z Hu J Wei N. Factors associated with mental health outcomes among health care workers exposed to coronavirus disease 2019. JAMA Netw Open. (2020) 3:e203976. 10.1001/jamanetworkopen.2020.3976 32202646 PMC7090843

[9] Li G Miao J Wang H Xu S Sun W Fan Y. Psychological impact on women health workers involved in COVID-19 outbreak in Wuhan: a cross-sectional study. J Neurol Neurosurg Psychiatr. (2020) 91:895–7. 10.1136/jnnp-2020-323134 32366684

[10] Lu W Wang H Lin Y Li L. Psychological status of medical workforce during the COVID-19 pandemic: a cross-sectional study. Psychiatry Res. (2020) 288:112936. 10.1016/j.psychres.2020.112936 32276196 PMC7195354

[11] Zhu Z Xu S Wang H Liu Z Wu J Li G. COVID-19 in Wuhan: sociodemographic characteristics and hospital support measures associated with the immediate psychological impact on healthcare workers. EClinicalMedicine. (2020) 24:100443. 10.1016/j.eclinm.2020.100443 32766545 PMC7311903

[12] Gloster AT Lamnisos D Lubenko J Presti G Squatrito V Constantinou M. Impact of COVID-19 pandemic on mental health: an international study. PLoS ONE. (2020) 15:e0244809. 10.1371/journal.pone.0244809 33382859 PMC7774914

[13] Ren Y Qian W Li Z Liu Z Zhou Y Wang R. Public mental health under the long-term influence of COVID-19 in China: geographical and temporal distribution. J Affect Disord. (2020) 277:893–900. 10.1016/j.jad.2020.08.045 33065831 PMC7444470

[14] Bethell J Aelick K Babineau J Bretzlaff M Edwards C Gibson JL. Social connection in long-term care homes: a scoping review of published research on the mental health impacts and potential strategies during COVID-19. J Am Med Dir Assoc. (2021) 22:228–37 e25. 10.1016/j.jamda.2020.11.025 33347846 PMC9186333

[15] Xinhua. The Number of COVID-19 Patients in Hospital in Wuhan Was Cleared. (2020). Available online at: http://www.xinhuanet.com/2020-04/26/c_1125908879.html

[16] Wang C Pan R Wan X Tan Y Xu L McIntyre RS. A longitudinal study on the mental health of general population during the COVID-19 epidemic in China. Brain Behav Immun. (2020) 87:40–8. 10.1016/j.bbi.2020.04.028 32298802 PMC7153528

[17] Tan YQ Wang Z Yap QV Chan YH Ho RC Hamid A. Psychological health of surgeons in a time of COVID-19: a global survey. Ann Surg. (2021). 10.1097/SLA.0000000000004775. [Epub ahead of print]. 33491983 PMC9762613

[18] Tee M Wang C Tee C Pan R Reyes PW Wan X. Impact of the COVID-19 pandemic on physical and mental health in lower and upper middle-income asian countries: a comparison between the Philippines and China. Front Psychiatry. (2020) 11:568929. 10.3389/fpsyt.2020.568929 33633595 PMC7901572

[19] Wang C Chudzicka-Czupala A Grabowski D Pan R Adamus K Wan X. The association between physical and mental health and face mask use during the COVID-19 pandemic: a comparison of two countries with different views and practices. Front Psychiatry. (2020) 11:569981. 10.3389/fpsyt.2020.569981 33033485 PMC7510452

[20] Asif IM Price DE Ewing A Rao AL Harmon KG Drezner JA. The impact of diagnosis: measuring the psychological response to being diagnosed with serious or potentially lethal cardiac disease in young competitive athletes. Br J Sports Med. (2016) 50:163–6. 10.1136/bjsports-2015-095560 26612845

[21] Kroenke K Spitzer RL Williams JB. The PHQ-9: validity of a brief depression severity measure. J General Internal Med. (2001) 16:606–13. 10.1046/j.1525-1497.2001.016009606.x PMC1495268 11556941

[22] Kroenke K Spitzer RL Williams JB Monahan PO Lowe B. Anxiety disorders in primary care: prevalence, impairment, comorbidity, and detection. Ann Internal Med. (2007) 146:317–25. 10.7326/0003-4819-146-5-200703060-00004 17339617

[23] Dumont M Provost MA. Resilience in adolescents: protective role of social support, coping strategies, self-esteem, and social activities on experience of stress and depression. J Youth Adolesc. (1999) 28:343–63. 10.1023/A:1021637011732

[24] Lai X Wang M Qin C Tan L Ran L Chen D. Coronavirus disease 2019 (COVID-2019) infection among health care workers and implications for prevention measures in a tertiary hospital in Wuhan, China. JAMA Netw Open. (2020) 3:e209666. 10.1001/jamanetworkopen.2020.9666 32437575 PMC7243089

[25] Chao M Xue D Liu T Yang H Hall BJ. Media use and acute psychological outcomes during COVID-19 outbreak in China. J Anxiety Dis. (2020) 74:102248. 10.1016/j.janxdis.2020.102248 32505918 PMC7255752

[26] Cuello-Garcia C Pérez-Gaxiola G van Amelsvoort L. Social Media can have an impact on how we manage and investigate the COVID-19 pandemic. J Clin Epidemiol. (2020) 127:198–201. 10.1016/j.jclinepi.2020.06.028 32603686 PMC7320665

[27] Pérez-Lugo M. Media uses in disaster situations: a new focus on the impact phase. Soc Inquiry. (2004) 74:210–25. 10.1111/j.1475-682X.2004.00087.x

[28] Freeman D Waite F Rosebrock L Petit A Causier C East A. Coronavirus conspiracy beliefs, mistrust, and compliance with government guidelines in England. Psychol Med. (2020) 4:1–13. 10.1017/S0033291720001890 32436485 PMC7264452

[29] Yao H. The more exposure to media information about COVID-19, the more distressed you will feel. Brain Behav Immun. (2020) 87:167–9. 10.1016/j.bbi.2020.05.031 32413557 PMC7215146

[30] Ghosh K. Violence against doctors: a wake-up call. Indian J Med Res. (2018) 148:130–3. 10.4103/ijmr.IJMR_1299_17 30381535 PMC6206759

[31] Sen M Honavar SG. It's a doc's life - workplace violence against doctors. Indian J Ophthalmol. (2019) 67:981–4. 10.4103/ijo.IJO_1166_19 31238390 PMC6611293

[32] Pieh C Budimir S Probst T. The effect of age, gender, income, work, and physical activity on mental health during coronavirus disease (COVID-19) lockdown in Austria. J Psychosomatic Res. (2020) 136:110186. 10.1016/j.jpsychores.2020.110186 32682159 PMC7832650

[33] Uutela A. Economic crisis and mental health. Curr Opin Psychiatry. (2010) 23:127–30. 10.1097/YCO.0b013e328336657d 20087188

[34] Xinhua. Chinese Pay Respect for Dedication of Medical Workers Amid Anti-Epidemic Battle. (2020). Available online at: http://en.people.cn/n3/2020/0226/c90000-9662252.html (accessed on February 26, 2020).

[35] Chen Q Liang M Li Y Guo J Fei D Wang L. Mental health care for medical staff in China during the COVID-19 outbreak. Lancet Psychiatry. (2020) 7:e15–6. 10.1016/S2215-0366(20)30078-X 32085839 PMC7129426

[36] Pierce M Hope H Ford T Hatch S Hotopf M John A. Mental health before and during the COVID-19 pandemic: a longitudinal probability sample survey of the UK population. Lancet Psychiatry. (2020) 7:883–92. 10.1016/S2215-0366(20)30308-4 32707037 PMC7373389

[37] Chen YC Kao CF Lu MK Yang YK Liao SC Jang FL. The relationship of family characteristics and bipolar disorder using causal-pie models. Eur Psychiatry J Assoc Eur Psychiatrists. (2014) 29:36–43. 10.1016/j.eurpsy.2013.05.004 23871494

[38] Chew NWS Ngiam JN Tan BY Tham SM Tan CY Jing M. Asian-Pacific perspective on the psychological well-being of healthcare workers during the evolution of the COVID-19 pandemic. BJPsych Open. (2020) 6:e116. 10.1192/bjo.2020.98 33028449 PMC7542327

[39] Lawal AM Alhassan EO Mogaji HO Odoh IM Essien EA. Differential effect of gender, marital status, religion, ethnicity, education and employment status on mental health during COVID-19 lockdown in Nigeria. Psychol Health Med. (2020) 6:1–12. 10.1080/13548506.2020.1865548 33351644

[40] Chew NWS Lee GKH Tan BYQ Jing M Goh Y Ngiam NJH. A multinational, multicentre study on the psychological outcomes and associated physical symptoms amongst healthcare workers during COVID-19 outbreak. Brain Behav Immun. (2020) 88:559–65. 10.1016/j.bbi.2020.04.049 32330593 PMC7172854

[41] Rattay P Michalski N Domanska OM Kaltwasser A De Bock F Wieler LH. Differences in risk perception, knowledge and protective behaviour regarding COVID-19 by education level among women and men in Germany. Results from the COVID-19 Snapshot Monitoring (COSMO) study. PLoS ONE. (2021) 16:e0251694. 10.1371/journal.pone.0251694 33979413 PMC8116045

[42] Lateef T Chen J Tahir M Lateef TA Chen BZ Li J. Typhoon eye effect versus ripple effect: the role of family size on mental health during the COVID-19 pandemic in Pakistan. Global Health. (2021) 17:32. 10.1186/s12992-021-00685-5 33781286 PMC8006139

[43] Le HT Lai AJX Sun J Hoang MT Vu LG Pham HQ. Anxiety and depression among people under the nationwide partial lockdown in Vietnam. Front Public Health. (2020) 8:589359. 10.3389/fpubh.2020.589359 33194995 PMC7658379

[44] Tran BX Nguyen HT Le HT Latkin CA Pham HQ Vu LG. Impact of COVID-19 on economic well-being and quality of life of the Vietnamese during the national social distancing. Front Psychol. (2020) 11:565153. 10.3389/fpsyg.2020.565153 33041928 PMC7518066

[45] Nguyen TTP Nguyen LH Le HT Vu GT Hoang MT Nguyen DN. Perceptions and attitudes toward COVID-19-related national response measures of Vietnamese: implications for pandemic prevention and control. Front Public Health. (2020) 8:589053. 10.3389/fpubh.2020.589053 33163473 PMC7581721

[46] Hao F Tan W Jiang L Zhang L Zhao X Zou Y. Do psychiatric patients experience more psychiatric symptoms during COVID-19 pandemic and lockdown? A case-control study with service and research implications for immunopsychiatry. Brain Behav Immun. (2020) 87:100–6. 10.1016/j.bbi.2020.04.069 32353518 PMC7184991

[47] Soh HL Ho RC Ho CS Tam WW. Efficacy of digital cognitive behavioural therapy for insomnia: a meta-analysis of randomised controlled trials. Sleep Med. (2020) 75:315–25. 10.1016/j.sleep.2020.08.020 32950013

[48] Moreno C Wykes T Galderisi S Nordentoft M Crossley N Jones N. How mental health care should change as a consequence of the COVID-19 pandemic. Lancet Psychiatry. (2020) 7:813–24. 10.1016/S2215-0366(20)30307-2 32682460 PMC7365642

[49] Chew NWS Cheong C Kong G Phua K Ngiam JN Tan BYQ. An Asia-Pacific study on healthcare workers' perceptions of, and willingness to receive, the COVID-19 vaccination. Int J Infect Dis. (2021) 106:52–60. 10.1016/j.ijid.2021.03.069 33781902 PMC7997703

[50] Deolmi M Pisani F. Psychological and psychiatric impact of COVID-19 pandemic among children and adolescents. Acta Bio Med Atenei Parmensis. (2020) 91:e2020149. 10.23750/abm.v91i4.10870 33525229 PMC7927507

[51] >Jiménez-Pavón D Carbonell-Baeza A Lavie CJ. Physical exercise as therapy to fight against the mental and physical consequences of COVID-19 quarantine: special focus in older people. Progress Cardiovasc Dis. (2020) 63:386–8. 10.1016/j.pcad.2020.03.009 32220590 PMC7118448

